# Introduction of day case hip and knee replacement programme at an inpatient ward is safe and may expedite shortening of hospital stays of traditional arthroplasties

**DOI:** 10.1186/s13018-021-02737-3

**Published:** 2021-10-11

**Authors:** Gyorgy Lovasz, Attila Aros, Ferenc Toth, John Va Faye, Marco La Malfa

**Affiliations:** 1 Practice Plus Group Hospital Barlborough, 2 Lindrick Way, Barlborough, S43 4XE Chesterfield UK; 2 The Royal Orthopaedic Hospital NHS Foundation Trust, Birmingham, UK

**Keywords:** Hip and knee arthroplasty, Day case, Shortening, Lengths of stay

## Abstract

**Purpose:**

We investigated the safety of primary hip and knee replacements with same day discharge (SDD) and their effect on length of stay (LOS) of traditional inpatient arthroplasties at our elective orthopaedic ward.

**Methods:**

200 patients underwent elective, unilateral primary day case total hip (THA, *n* = 94), total knee (TKA, *n* = 60) and unicondylar knee replacements (UKA, *n* = 46). SDD rates, reasons for failure to discharge, readmission, complication and satisfaction rates were recorded at 6-week follow up. Changes in LOS of inpatient arthroplasties (*n* = 6518) and rate of patients discharged with only one night stay treated at the same ward were tracked from 1 year prior to introduction of day case arthroplasty (DCA) program to the end of observation period.

**Results:**

166 patients (83%) had SDD while 34 (17%) needed overnight stay. Main reasons for failure to discharge were lack of confidence (4%) fainting due to single vasovagal episode (3.5%), urine retention (3%) and late resolution of spinal anaesthesia (3%). 5 patients (3%) had readmission within 6 weeks, including 1 (0.6%) with a partial and treated pulmonary embolism. 163 patients were satisfied with SDD (98%). After launching the DCA program, average LOS of inpatients was reduced from 2.3 days to 1.8 days and rate of discharge with only 1-night stay increased from 12% to around 60%.

**Conclusion:**

Introduction of routine SDD hip and knee arthroplasty programme at an elective orthopaedic centre is safe and also may confer wider benefits leading to shorter inpatient hospital stays.

## Introduction

Outpatient hip and knee arthroplasty with same day discharge (SDD) gains popularity worldwide. This has become widespread practice in the USA and an abundance of literature shows that the practice is associated with high satisfaction, low complication rates and is a safe and feasible option for selected patient population [1–6]. Encouraging outcomes have also been reported from European centres albeit in smaller cohorts of patients [7–14]. Benefits of SDD are not limited to clinical outcomes but also advantageous to the healthcare systems especially in resource stressed environments by freeing up capacity and offering significant cost savings [15]. SDD arthroplasty can be performed at dedicated Ambulatory Surgical Centres (ASC), hospital outpatient surgery departments (HOSD) and at hospital inpatient wards. The safety of DCA on selected patient population is well documented in all these settings [14, 16, 17]. Recent publications comparing the inpatient versus outpatient settings concluded that the latter is more time efficient and patients are more likely to achieve discharge criteria on the day of surgery [14, 18]. Although outpatient-based arthroplasty may offer these benefits, the hospital-ward approach provides a safe solution for those patients who are unable to discharge. After considering these opinions, we introduced our SDD program within a mixed inpatient setting on a selected cohort of patients at our high-volume orthopaedic centre in 2018. Planned SDD patients were treated at our orthopaedic ward mixed with traditional inpatient arthroplasties. As we made successful progress with day cases, we observed a concomitant reduction of LOS in our standard inpatient pathway. Thus far, scientific literature pertinent to such an impact of DCC on standard joint replacement patients has not been reported.

The paucity of these data was the main motivation for undertaking the present analysis of the associated impact on the inpatient pathway in addition to assessing patient safety features of day cases.

## Patients and methods

Standard Operating Procedure was developed for day case arthroplasties and approved by Clinical Governance structure of the hospital.

200 patients were selected for unilateral total hip (THA, *n* = 94), total knee (TKA, *n* = 60) and unicondylar knee arthroplasties (UKA, *n* = 46) with planned SDD based on a pertinent inclusion criteria (Table 2) between March 2018 and December 2020. From the high number of patients having no exclusion criteria for SDD we selected those exhibiting strong motivation for same day discharge. Patients had the same standard pre-operative assessment as inpatients but once consenting to be a potential day case, they were educated in SDD pathway. Perioperative data were collected prospectively. Demographic data and selection criteria are presented in Tables 1 and 2.

**Table 1:** Demographic and ASA data of 200 patients admitted for day case arthroplasty (DCA)

Parameter	THA	TKA	UKA
Male (*n*)	67	41	28
Female (*n*)	27	19	18
Age (mean, range)	63.1 (46–81)	67.4 (48–82)	62.3 (47–78)
BMI (mean, range)	27.6 (19.8–38.7)	30.6 (21.8–39.2)	30.2 (20.3–38.7)
ASA grade I (*n*)	34	11	9
ASA grade II (*n*)	60	49	37

*THA* total hip arthroplasty, *TKA* total knee arthroplasty, *UKA* unicondylar knee arthroplasty, *AS*A American Society of Anestheziologists

**Table 2:** Patient selection criteria for day case arthroplasty (DCA)

Inclusion criteria	Exclusion criteria
ASA grade I and II	Type I diabetes
Strong motivation to go home same day	Chronic Obstructive Pulmonary Disease
Adequate postoperative home support	History of prostate pathology
Independent preoperative mobility status	Coronary artery disease
	Congestive heart failure
	Cirrhosis
	History of cerebrovascular or venous thromboembolic event
	Preoperative haemoglobin < 130 g/L
	Coagulopathies
	Anticoagulation therapy
	Cognitive disorders/dementia
	Dependent mobility status
	Lack of home support
	Chronic Kidney Disease 3 and above

*ASA* American Society of Anestheziologists

Patients were admitted on the day of surgery directly to the inpatient ward and were operated on as first or second on the list with a cut off surgery end-time at 13.00 h. An established enhanced recovery protocol was followed and a trained team of nurses and physiotherapists were allocated to manage their care.

The standard hospital protocol of spinal anaesthesia for arthroplasties was slightly modified for day cases. Short acting, opiate free formula (50–60 mg Prilocaine hydrochloride 2%) was used to accommodate early mobilization and reduce the risk of urine retention. One patient requested general anaesthesia. Ultrasound guided Hunter`s canal (adductor canal) block was performed for knee replacements utilising 8–10 ml 0.5% levobupivacaine [19, 20]. Postoperatively, patients were observed for a short period of time in theatre recovery before being transferred to the orthopaedic ward.

The surgical technique, mechanical and chemical venous thromboembolism (VTE) prophylaxis, peri-operative antibiotic regime and tranexamic acid protocol was identical to inpatient arthroplasties. Local infiltration analgesia (LIA) was used in both hip and knee arthroplasties. Patients were mobilized as soon as short acting spinal anaesthesia wore off via the same enhanced, unrestricted physiotherapy protocol and fully weight bearing as on the inpatient pathway. For planned day cases, intervals between consecutive steps of mobilisation were shortened depending on medical condition and tolerance of the patient to facilitate achievement of discharge criteria by the end of the day.

Postoperative radiographs were obtained and checked before patients were released to home with a cut off at 19:00 h. Discharge criteria were identical to traditional, inpatient hip and knee replacements i.e. stable vital signs, controlled post-operative pain, absence of urine retention, independent transfer in and out of bed, capability to independently ascend and descend one flight of stairs and for knee arthroplasties, sufficient flexion and good quadriceps activation for safe ambulation. A 24 h per day seven days per week manned telephone number was provided. Take home medication (Paracetamol, Codeine Phosphate, Ibuprofen, Omeprazole) including rescue painkillers (Oxycodone Hydrochloride 5–10 mg prn) for 7 days were supplied. Postoperative follow up phone call was arranged for the following morning. Patients received community physiotherapy but also had access to hospital physiotherapy in case of any setbacks in recovery. As it has been reported that the vast majority of early complications occur within 6 weeks postoperatively [16], we maintained the standard 6-week arthroplasty follow up protocol of our hospital which in place for all joint replacements. In addition to clinical assessment, satisfaction rating with SDD was obtained, i.e. satisfied and would undergo DCA again, overnight stay would have been better or dissatisfied. Alternative provider hospital or emergency department attendances or readmissions, medical and surgical complications (e.g. VTE or cardiovascular events, dislocation, surgical infections, reoperation) were recorded.

We carried out the retrospective analysis of the LOS and the rate of next day discharges of inpatient arthroplasties (*n* = 6518) starting 1 year prior to launch of DCAs to the end of observation period for any relevant trend-changes in hospital stays on traditional inpatient pathway. Correlation coefficient (Pearson`r) was calculated between the number of day cases performed and decrease of LOS and increase of rate in 1-night stay discharges of inpatient arthroplasties in the same time period. *R* > 0.5 or <  − 0.5 was set for strong correlation.

## Results

**Fig. 1:**
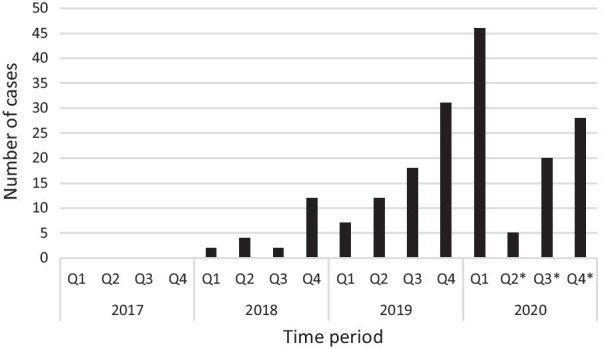
Number of day case arthroplasties (DCA) performed in 2017–2020 (*Q* quarter, * Covid-19 capacity restrictions)

75 patients had uncemented and 19 received hybrid THA while 60 patients received cemented TKA and 46 medial, cemented fixed bearing UKA. Number of DCAs performed over the study period are presented in Fig. 1.

### Same day discharge (SDD) rate

Out of 200 patients booked as day case, 166 went home the same day (83%). Knee arthroplasties had better SDD rates than hips (Table 3). All but two of overnight stayers went home the next day. Details and reasons for overnight stays are listed in Table 4.

**Table 3:** Same day discharge (SDD) rates of 200 hip and knee arthroplasties

Surgery	Booked (*n*)	SDD (*n*)	Rate (%)
THA	94	72	76
TKA	60	53	88
UKA	46	41	89
Total	200	166	83

*THA* total hip arthroplasty, *TKA* total knee arthroplasty, *UKA* unicondylar knee arthroplasty

**Table 4:** Reasons for unplanned overnight stays of day case arthroplasties (DCA)

Reasons	THA (*n*)	UKA (*n*)	TKA (*n*)	Total (*n*)
Lack of confidence or patient choice	5	1	2	8
Fainting (vasovagal)	7	0	0	7
Urinary retention	4	2	0	6
Late resolution of spinal	3	1	2	6
Tachycardia	2	0	1	3
pain management	1	0	0	1
Wound ooze	0	0	2	2
Hypertension	0	1	0	1
Total	22	5	7	34

*THA* total hip arthroplasty, *TKA* total knee arthroplasty, *UKA* unicondylar knee arthroplasty

### Readmissions, complications

No patients were lost to follow up, all readmissions and complications were recorded. Five readmissions occurred within 6-weeks (3%). One THA for minor oozing from surgical wound, two TKA patients with suspected but unconfirmed surgical site infection (SSI) and with cellulitis on lower leg and one UKA for poor pain control. One TKA patient was readmitted with partial, treatable pulmonary embolism 7 days postoperatively (0.6%).

### Satisfaction rate

Three patients would have preferred overnight stay while 163 were satisfied and would undergo DCA again (98% satisfaction rate).

### Inpatient arthroplasty rates

**Fig. 2:**
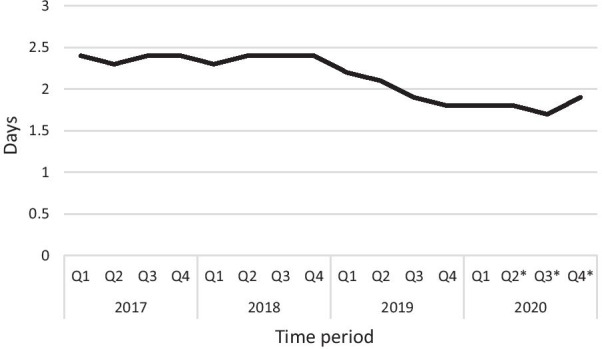
Average length of stay (LOS) of inpatient arthroplasties in 2017–2020 (*Q* quarter, * Covid-19 capacity restrictions)

**Fig. 3:**
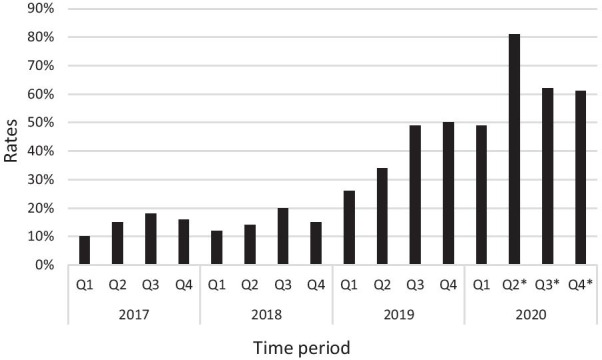
Changes in ratio of inpatient arthroplasties discharged with 1-night stay in 2017–2020 (*Q* quarter, * Covid-19 capacity restrictions)

Rate of 1-night stay of inpatients increased to around 60% from 14% prior to DCA within a few months after achieving high case numbers and this discharge rate was sustained to the end of observation period. The average LOS of inpatient arthroplasties reduced to 1.8 days from 2.3 days (22% reduction) in the same time span (Figs. 2 and 3). Correlation coefficient calculation showed strong correlation between number of day cases performed and reduction of LOS (*r* =  −0.86) and increase of 1-nigh stays (*r* = 0.83) of inpatient arthroplasties.

## Discussion

In the selection process, choosing patients with appropriate motivation for early discharge is essential [6, 21, 22]. Lack of awareness and low level of confidence in DCA within the patient cohort populations has been shown to be a barrier to allocate otherwise eligible patients to SDD [23]. Our experience was consistent with these findings, high proportion of potential hip and knee replacement patients were unaware of and reluctant to accept the day case option when seen at pre-operative assessment. For this reason, we only selected those from medically eligible patients for the DCA pathway who showed strong motivation and family support to go home the same day.

The overall SDD rate was 83% and knee arthroplasties had higher rates (88% for TKA and 89% for UKA) than THAs (76%). Postoperative anaemia and need of blood transfusion after joint replacements are usually among the main causes of delayed hospital discharges [24]. Patients in our series were optimized for preoperative haemoglobin, intraoperative blood loss was minimized by tranexamic acid, thorough haemostasis and short operating time. As a result, postoperative anaemia was not a reason for failure to discharge the same day. This finding is consistent with literature reports concluding that perioperative blood management plan should be integral part of fast-track pathways and is a key factor in the success of early discharges [25]. Postoperative pain was well controlled in each group and was an uncommon reason of failure to discharge (1 patient only). This observation is somewhat contradictory to previous publications which have reported pain and lack of mobilization to be significant obstruction to early discharge of knee replacements [26, 27]. Potential explanation for this difference may be that these studies did not use adductor (Hunter`s) canal block for knee arthroplasties which was part of our protocol. It resulted in excellent pain control without motor deficit and allowed for early mobilization. The most important medical reason for overnight stays was orthostatic hypotension exclusively in THA group which is a well-documented untoward event during postoperative mobilization of hip arthroplasties [28, 29]. The lack of this phenomenon in cases of knee arthroplasties may be another factor that explains better SDD rate of knees in our series in contrast to previous studies [8, 30].

Our SDD rates are in line with the published rates of unplanned overnight stays which range from 75 to over 99% [31–34].

Readmissions comprised 3% of DCAs consistent with recent European studies [16] as well as with reports published on large overseas patient cohorts [4, 17, 35–37]. There was only one readmission (0.6%) due to a serious complication; a TKA patient with partial pulmonary embolism 7 days after index procedure. This patient had been mobilized per protocol, had no preoperative risk factor for VTE and our root cause analysis could not identify association with SDD. Within 24 h, we had one readmission for wound ooze while the rest of hospitalisations were unrelated to SDD as similar causes, like suspected SSI, commonly warrant repeated admissions with inpatient arthroplasties as well [38].

In addition to low readmission and complication rates, day cases were associated with very high satisfaction rates (98%) which indicates that our outcomes are consistent with the overwhelming majority of reported case series and meta-analysis and support the opinion that DCA is feasible, safe and highly satisfactory option for suitable patient cohorts in both outpatient and inpatient settings [3, 12–14, 16, 34, 39, 40].

Another affirmative consequence of DCAs treated in the arthroplasty ward was the shortening of LOS over the entire cohort of patients within the traditional inpatient pathway. Shortening of hospital stay with arthroplasties has been a general trend worldwide [41, 42], but the observation of a rapid, simultaneous reduction in LOS with the increase of DCA patient cohort has not been reported before. Prior to introduction of SDD and during the low volume pilot phase, rate of 1-night stays was constantly approximating 15%, with inpatient LOS at 2.3 days. In parallel with the surge of DCA numbers, fast increase of next day discharges to 60% and decrease of LOS to 1.8 days on the inpatient pathway was noticed and maintained during the entire observation period even when day case numbers dropped considerably because of Covid capacity restrictions (Figs. 1, 2 and 3). The strong correlation was verified by Pierson coefficient as well. Outpatient-based arthroplasty has been reported more time efficient, patients spend about 30% less time in the unit compared to day cases treated at an inpatient ward. Apart from differences in comfort conditions and the higher staff-patient ratio, this is generally attributed to attentive teams working fully focused toward early discharge at a separated outpatient department [14, 18]. In our ward set up, we assigned dedicated but non-exclusive staff to treat day cases and eventually our entire ward team became skilled in and confident with the day case rehabilitation protocol. The inevitable split of attention of shared medical and physiotherapy teams may have slightly extended hospital stay of day cases with a few hours but appeared to boost earlier discharges on the inpatient pathway. As the main differences in postoperative rehabilitation were swifter mobilization, more intense and time-sensitive medical observation of day cases, we hypothesize that success of SDD protocol inspired the ward teams to adopt similar practices on the inpatient pathway thus helping medically fit patients achieve discharge criteria earlier. On the other hand, a larger subset of inpatients may have felt empowered to engage with fast-track regime by witnessing first-hand the rapid progression of day cases. It appears that supplementing our physiotherapy and medical team skills with day case management routine has improved their efficacy and contributed to acceleration of inpatient recovery.

We believe that this pleiotropic effect is an important observation as it demonstrates that in addition to significant financial benefits of DCA alone [15], the shortening of inpatient stay may offer further cost savings and capacity relief for hospitals where significant number of beds are reserved for elective arthroplasties.

Strength of our study is that, compared with the European standards, our case numbers are high, thus adding to the literature data regarding safety of SDD arthroplasty. Our study presents a new finding namely, that treating SDD patients mixed with traditional arthroplasties in the same ward may facilitate shorter hospitalisation of inpatients.

This study has weaknesses including lack of matched inpatient group and longer than 6 weeks follow up with validated outcome measures.

## Conclusions and novelty

In summary we conclude that SDD hip and knee replacements for selected patients can safely be introduced in an orthopaedic ward with established fast-track protocol and as an additional benefit, LOS of traditional inpatient arthroplasties may reduce.

## Data Availability

Corresponding authors confirms that summary of all data are included in the manuscript. Details are available upon request.

## Abbreviations

ASC: Ambulatory Surgical Centre
ASA: American Society of Anestheziologists
BMI: Body mass index
DCA: Day case arthroplasty
HOSD: Hospital outpatient surgery department
SDD: Same day discharge
LIA: Local infiltration analgesia
LOS: Length of stay
SSI: Surgical site infection
THA: Total hip arthroplasty
TKA: Total knee arthroplasty
UKA: Unicondylar knee arthroplasty
VTE: Venous thrombembolism
Q: Quarter
